# Editorial: Case reports in thrombosis: 2024

**DOI:** 10.3389/fcvm.2025.1614440

**Published:** 2025-05-01

**Authors:** Luca Spiezia

**Affiliations:** First Chair of Internal Medicine, Department of Medicine, Padova University Hospital School of Medicine, Padova, Italy

**Keywords:** venous thromboembolism, anticoagulants, case report, risk factors, thrombophilia

**Editorial on the Research Topic**
Case reports in thrombosis: 2024

This editorial presents a collection of articles published in Frontiers in Cardiovascular Medicine: Case Reports in Thrombosis: 2024. The manuscripts collected in this Research Topic are characterized by a wide variety of topics from genetic risk factors of venous thromboembolism to the management of rare and potentially fatal comorbidities. We hope that the reader will obtain vital insights for daily clinical practice. We would like to thank all the reviewers and the Editorial Office staff for their invaluable contribution. The following are the articles in the Frontiers in Cardiovascular Medicine: Case Reports in Thrombosis: 2024.

*Pulmonary artery in situ thrombosis due to patent ductus arteriosus: a case report*, by Wang et al.

Pulmonary artery *in situ* thrombosis (PAIST) is a rare condition and it refers to a thrombus originating in the pulmonary arterial tree, independently of peripheral thrombosis (1). Clinicians often misdiagnose PAIST as pulmonary embolism (PE) as both conditions manifest as low-density filling defects on computer tomography angiography. This case describes a healthy 59-year-old male hospitalized due to sudden onset of dyspnea with no fever or chest pain, suggesting PE. The patient received anticoagulation and antibiotics for a staphylococcus infection. A reevaluation of imaging revealed the pulmonary obstruction was in proximity to a patent ductus arteriosus (PDA), thus confirming the diagnosis of PAIST. The patient underwent open-heart surgery for PDA ligation and resection of the thrombus; he recovered well and was discharged with a prescription of digoxin and oral rivaroxaban. The authors underscore the importance of considering differential diagnoses for PE—and PAIST in particular—in patients with a single pulmonary lesion and no evidence of peripheral thrombosis.

*Case report: Madelung disease with postoperative priapism and multiple venous thromboses*, by Guo et al.

Madelung disease is a rare, benign metabolic disease of unknown pathogenesis characterized by symmetrical subcutaneous fat deposits throughout the body (2). Treatment consists mainly of lipectomy or liposuction. This case describes a 49-year-old male affected by type II Madelung disease with bilateral fat deposits in thighs, who developed unexplained priapism and multiple venous thromboses following each of four liposuction, and subsequent skin and fasciocutaneous flap surgeries. Continuously adjusted anticoagulation improved the patient's coagulation profile. Postoperative hypercoagulability is rare in Madelung disease, nevertheless, the authors highlight the importance of a thorough preoperative assessment of the thrombotic risk and constant postoperative monitoring to improve prognosis and outcomes.

*A case of Rapamycin-eluting stent for the treatment of refractory stenosis of arteriovenous fistula stenosis*, by Xiong et al.

An autologous arteriovenous fistula (AVF) is the preferred vascular access for patients who require hemodialysis. The most recurrent complication of AVF is stenosis and restenosis following recanalization via percutaneous transluminal angioplasty (PTA) or bare metal stent placement. The authors describe a 51-year-old female with end stage chronic kidney disease undergoing hemodialysis since 2014 via autologous AVF. The patient was repeatedly hospitalized for stenosis of the autologous AVF which was treated by PTA. Ultimately, another PTA was performed and an ultrasound-guided rapamycin-eluted stent placed; the vascular access did not develop another stenosis or thrombosis for a record 14 months, until the stent collapsed. Rapamycin inhibits smooth muscle cell proliferation and obstructive arteriopathy. The authors offer a potentially lifesaving therapeutic measure to preserve vascular accesses in patients undergoing dialysis prone to refractory stenosis.

*Case Report: Ectopic pulmonary embolism as a complication of bronchial artery embolization*, by Liu et al.

Bronchial artery embolization (BAE) is the first-line treatment in patients with massive hemoptysis (3). The present case describes a 59-year-old male with multiple congenital bronchial artery-pulmonary artery fistulas who was admitted due to acute hemoptysis. Treatment with hemocoagulase and vitamin K1 was unsuccessful, and an emergency BAE was performed due to sudden massive hemoptysis. The patient was readmitted two weeks later due to recurrent hemoptysis. Computed tomography pulmonary angiography showed pulmonary embolism treated with therapeutic low-molecular-weight heparin and percutaneous catheter-directed embolectomy. Hemoptysis resolved within 24 h and dyspnea improved significantly; he was discharged with a 3-month prescription of oral rivaroxaban. Histological analysis revealed that embolization particles entered the pulmonary circulation via an occult fistula masked by multiple bronchial artery branches. The 12-month follow-up showed no recurrences of dyspnea or hemoptysis. It is vital to thoroughly identify all abnormal communications — atypical fistulas, anastomoses — between the pulmonary and bronchial circulations and select appropriate embolization material during BAE.

*Case Report: IVC-agenesis and FVL mutation; successful DVT/PE treatment with direct oral anticoagulation (Factor Xa inhibitor)*, by Siddiqui et al.

Inferior vena cava (IVC) agenesis is a rare congenital malformation wherein collateral veins develop to maintain adequate blood flow to the heart from the lower extremity. This compensatory mechanism increases significantly the risk of venous thromboembolism (VTE), including deep vein thrombosis (DVT) and pulmonary embolism (PE). Heterozygous factor V Leiden is an inherited thrombophilia that carries a six- to eightfold increased risk of VTE (4). This case describes a 28-year-old obese male presenting with right lower extremity swelling and pain. Laboratory analyses and imaging revealed FVL and IVC agenesis, and the patient was diagnosed with extensive right extremity DVT by Doppler ultrasound. He received intravenous heparin and later switched to oral apixaban upon discharge. Indefinite anticoagulation with a well-tolerated oral anticoagulant remains the most effective strategy to mitigate the risk of recurrent thrombosis.

*Case Report: PROS1 (c.76 + 2_76 + 3del) pathogenic mutation causes pulmonary embolism*, by Ding et al.

Venous thromboembolism is a life-threatening condition that can be caused by several genetic disorders (5). Protein S is an essential natural anticoagulant whose deficiency results in hypercoagulability (6). This study reports the case of a 28-year-old male who presented with hemoptysis and right chest pain, who was diagnosed with acute pulmonary embolism and pneumonia. After receiving oxygen therapy and anticoagulation therapy, the patient underwent thrombus aspiration and thrombolysis. Genetic testing revealed a heterozygous mutation of protein S. Albeit extremely rare, inherited thrombophilia linked to PROS1 mutations may cause pulmonary embolism in subjects in apparent good health and with no obvious risk factors. Thrombolytic therapy and anticoagulation were effective in this patient.

*Perioperative management of caesarean section for a pregnant woman with Sjögren's syndrome and pulmonary embolism: A case report*, by Liu et al.

Sjögren's syndrome (SjS) is a chronic inflammatory autoimmune disease that affects predominantly women, whose complexity and complications increase significantly during pregnancy (7). This study describes a 37-week pregnant woman with SjS who presented with chest tightness and suffocation. She was hospitalized and scheduled for an emergency caesarean section due to low oxygen saturation and fetal distress. Sjögren's syndrome renders perioperative anesthesia management quite challenging. Furthermore, SjS can promote a hypercoagulable state. After a safe delivery, the patient was diagnosed with pulmonary embolism and lower extremity deep vein thrombosis. The authors highlight that pregnant women with SjS should receive critical care focused on maintaining fluid balance, continuous oxygen therapy and anticoagulation.

*Rare and life-threatening iliac vein stent infection following radiotherapy*, by Liao et al.

Radiotherapy (RT) may cause major tissue damage and other severe complications. This case describes a 43-year-old female who received multiple sessions of RT following cervical cancer surgery, and developed severe complications from improper iliac vein stent placement. Two months after completing RT, the patient presented with right lower extremity edema and pain. She was diagnosed with a compression of the right common iliac vein, thus prompting a stent placement. Over a month later, the patient was rehospitalized with high fever, recurrent right lower extremity swelling and severe pain. An exploratory laparotomy revealed a large abscess in the right iliac fossa, a colonic fistula with fecal leakage, and occluded iliofemoral stents. The stents were promptly removed, the veins ligated and the fistula repaired with a colostomy. Clinicians should thoroughly investigate common post-RT symptoms which may obscure underlying conditions. Preoperative evaluations for stent placement should include CT scans, especially in cancer patients, to accurately assess pelvic and abdominal anatomy.

*Successful management of coagulation dysfunction in a patient with fulminant myocarditis: A case report*, by Dong et al.

Fulminant myocarditis (FM) is a life-threatening condition predominately caused by viral infections and characterized by abrupt onset, diffuse myocardial inflammation and rapid clinical deterioration (8). Direct virus- and immune-mediated damage to myocardial cells may result in extensive myocardial dysfunction and impaired contractility, culminating in cardiogenic shock. This case report describes the case of a 51-year-old male hospitalized for intermittent abdominal distension, breathlessness and fatigue. Laboratory analyses showed antibodies to Coxsackievirus B3 and echovirus. The clinical management of the patient focused on three main areas: improvement of organ function, correction of coagulation disorders, and control of inflammation and infection. This case highlights the difficulty of managing a patient with FM complicated by cardiogenic shock, respiratory failure, severe liver injury and coagulopathy. It is essential to identify the underlying etiology of FM and implement a comprehensive therapeutic approach to improve outcomes.

*Case Report: Maintaining a balance between vascular access patency and stable dissection status in a hemodialysis patient with unrepaired type A aortic dissection*, by Lai et al.

Type A aortic dissection is a lethal condition wherein a tear develops within the vascular wall, treated surgically. This peculiar case describes a 72-year-old male undergoing hemodialysis since 2019 and diagnosed in 2017 with unrepaired extensive type A aortic dissection. The patient was admitted in 2022 due to a first episode of dialysis catheter occlusion which was resolved by urokinase thrombolysis. In the following eight months, the patient experienced four additional episodes: 2 resolved by urokinase thrombolysis and 2 treated by catheter replacement. The patient ultimately requested LMWH during hemodialysis, which presented a challenge for the clinicians due to the absence of standardized guidelines for the use of heparin in patients with unrepaired type A aortic dissection. Clinicians administered a lower dose of 1,000 U of intravenous LWMH which was gradually increased till a maximum of 2,000 U, combined with constant imaging surveillance of the dissection. This allowed the patient to safely undergo about four hours of hemodialysis each time for 25 consecutive months without experiencing further catheter occlusion and the dissection remained stable. The authors underscore the rarity of a patient living a normal life for over seven years with an unrepaired extensive aortic dissection. They hypothesized that it may due to the patient's extremely well-controlled blood pressure—hemodialysis-related dehydration and antihypertensive medication—and a spontaneous thrombosis occluding the dissection.
